# National Pension Fund for Nurses

**Published:** 1889-10-19

**Authors:** 


### National Pension Fund for Nurses.

The second general meeting of the members of the National Pension Fund for Nurses was held on Tuesday, at the offices, 8, King-street, Cheapside, E.C., Mr. Walter H. Burns (the chairman) presiding.

The Secretary (Mr. G. P. Pocock) read the notice convening the meeting, and also the minutes of the last general meeting, which were confirmed. The report and accounts having been presented and formally received,

The Chairman said: It now becomes my duty to move that the various accounts, report, and balance-sheet be adopted, and entered upon the minutes. In submitting the report, which is now before you in a printed form, there is very little left for me, as chairman, to add, as the information contained therein is so full. As, however, this is the first meeting at which any tangible results could be presented to the members of the Pension Fund, it may not be inappropriate for me to state that, up to the present, the results have far exceeded the expectations of the council, or even of those of the generous promoters who started the good work. That we should have been able in the first year of our practical working to get all the expenses paid, enrol upwards of 1,000 nurses under our banner, and invest for their benefit a sum amounting to upwards of £40,000, including the annuity and general accounts, is a result which certainly is most gratifying. It is all the more so when we consider that it has been obtained by the earnest co-operation of a few men, and of the nurses themselves, without any advertising or appeal to the general public. Our present position is such that there is no doubt whatever that the "loading" upon our premiums will pay all the very moderate expenses of conducting the business, and that the interest upon the entire donation and bonus funds, in addition to the capital itself, can thus be applied towards increasing the insurance bonuses to the nurses. We should not, however, by any means rest content with this. The general public will, no doubt, respond most freely to an appeal to them for so good a work when once they see that it is fairly established beyond risk of failure, and in the hands of people in whom they have confidence. I strongly recommend that such an appeal should be made. In the first place, it will be a great stimulus to the nurses to join in large numbers if they feel that the fund will thus materially supplement the payments which they themselves are able to afford. The donations can take the form either of a lump sum paid to the bonus fund, or of an annual subscription for a certain number of years. Should any individual desire to benefit a particular nurse who may have attended to him or his family, or to whom he feels some especial gratitude, the council could open a special account for the amount which he may so give, and apply the sum for the benefit of the particular nurse whom he may indicate. It is a matter of admiration to all the council to see what a spirit of saving and prudent forethought has been made evident by the working of the fund, and how splendidly the nurses themselves have co-operated, out of their usually very slender means, in securing a provision for their declining years. One of the great merits of our fund is that it is in no sense a charitable institution. While it is conducted almost free of expense, and the council take care that the funds are wisely invested, it only benefits those who are willing to help themselves by their own self-sacrifice, economy, and thrift. It is needless for me to point out all the claims which the nurses as a body have upon the public. While institutions for sailors and soldiers are numerous, and provisions for workers in almost every field are made on every hand, the nurses alone, who are daily risking their lives to save those of the community at large, and whose heroism and self-sacrifice are surpassed in no other profession, have not as yet had an adequate reward from the public, and I feel sure that it has only to be properly brought before them to bring in a large contribution to the bonus fund. In conclusion, I cannot refrain from expressing the gratitude of the council and of a who are interested in the general welfare of nurses, for the willingness shown by the Prince and Princess of Wales to identify themselves with the National Pension Fund, thereby not only testifying to its intrinsic merits, but giving another proof of the continuous watchfulness they exercise over the development of every movement for the social improvement of the people.

Dr. J. C. Steele seconded the resolution, which was unanimously adopted.

Mr. Henry C. Burdett (deputy-chairman) said the chairman had thrown out the idea that it would be desirable, as the nurses had come forward and shown how thrifty and self-denying they could be, that they should offer the general public an opportunity of recording their appreciation of those efforts in a substantial way. He (Mr. Burdett) therefore desired to move "That the council be requested to take immediate steps to increase the donation bonus fund to as great an extent as possible." He believed that this fund would meet the wishes of very many earnest and good people all over the country. It was now quite twenty years since Mr. Homer Chance, of Birmingham, said, "If you can establish a pension fund for nurses I will give £500 to a bonus fund." For that he had never asked, because he had never been in a position to redeem his promise until now, but he had no doubt that it stood good, and that Mr. Chance would welcome the opportunity of showing his appreciation of the work of nurses by subscribing to the bonus fund in connection with the National Pension Fund for Nurses. There were two aspects in which he thought this matter might be considered. First of all, they were not in a position before that day, because the fund was not legally constituted, to ask the public to come forward and give to the donation bonus fund, although, from spontaneous donations, it now amounted to nearly £30,000. As they were now in this position, he thought it right and desirable that the public and all who were interested in the nurses should understand what the bonus fund would do. First of all, it would confer a great benefit on the public. He knew himself from the experience he had had in the working of hospitals, that when a gentleman in the same station of life as themselves recovered from illness, and attributed it in no small degree to the care of the nurses, in the day of convalescence he was puzzled to know how he could show his gratitude properly. The usual form had been to give some present to the nurse, a present of a perishable character which often cost a substantial sum, and which really did very little good. One nurse of his acquaintance received six work-boxes in the same year from grateful patients. Now this fund would supply a means of meeting this want in the case of those who felt indebted to the nurses wherever they might be, because the council had arranged that anyone could pay a sum of money to the credit of any particular nurse, to be available when she was too old or too ill to work and required to be looked after herself. He thought the donation bonus fund would do a very great deal of good, and be very widely appreciated for that reason. Then, so far as the nurses themselves were concerned, experience had shown that they could not, unaided, out of their limited wages, provide an adequate pension for themselves. The knowledge of this fact only required to be widely spread to ensure a liberal response to the appeal for the donation bonus fund in connection with the National Pension Fund for Nurses. Although this National Pension Fund was that day established in a strong position, and would, no doubt, go forward and have a very great future, it would not have been possible for it to have attained its present dimensions except its work, its necessity, and the earnestness of its promoters had been recognised from the outset by the Press. He was sure he was only expressing the views of the council when he said that they were greatly indebted to the Press, because the cordial co-operation of the editors had reduced their advertising expenses to almost nothing, and in one year they had been able to transact a very large amount of business in connection with the Pension Fund. If the

Press that day explained what the bonus fund proposed to do he was perfectly certain it would not be necessary to beg for money, and all that was required would be forthcoming without difficulty or delay.

Mr. Clifford Wigram seconded the resolution, which was cordially agreed to; on the motion of the chairman, seconded by Mr. Edward Rawlings, the auditor (Mr. Frederick Whinney) was reappointed, the meeting at the same time expressing its gratitude and appreciation of his care, industry, and liberality in looking after the interests of the association.

Mr. Wigram said the fund never could have been started without a deposit of £20,000. Four gentlemen very liberally came forward and lent the money. Since then they had very generously converted their loan into a gift, and one of them, Mr. Junius S. Morgan, had given another donation of £5,000 (applause). Several other donations had been given—to the bonus fund £25,667, and for preliminary expenses extending over a long period, which otherwise must have come out of the general fund, £1,287, consisting of £500 from Mr. Henry C. Burdett, £262 10s. from Mr. E. P. Coates, and £525 from Messrs. Murietta and Co. He did not think it would be right to separate without according their hearty thanks to those gentlemen who had come forward and assisted them in starting what he hoped would be a substantially good work. He therefore moved "That the best thanks of the meeting be given to Lord Rothschild, Messrs. Anthony Gibbs and Sons, Mr. E. A. Hambro, Mr. Junius S. Morgan, and the other gentlemen who have so liberally come forward and given donations towards the funds of this society."

Mr. Percival A. Nairne, Deputy-Chairman of the Seamen's Hospital, seconded the resolution, which was cordially adopted.

Mr. Burdett proposed a vote of thanks to the chairman, remarking that it was really to him, indirectly, as the representative of their good friend Mr. Junius S. Morgan, who had always stood by them, that the fund owed its existence. Mr. Burns had rendered invaluable service in making their investments, as the report proved.

Dr. Steele seconded the motion, which was agreed to.

The Chairman acknowledged the compliment, and the proceedings terminated.

